# Dr. Flint to the Subscribers of the Buffalo Medical Journal

**Published:** 1860-03

**Authors:** Austin Flint


### EDITORIAL DEPARTMENT.

*To the Subscribers of the New York Monthly Review and Buffalo Medical Journal.*—A change in the management of an old and firmly established journal should be a matter of regret, if it be not productive of decided benefit to the subscribers, and through them, to the conductors of the journal. We considered that we were making such a change when we engaged a business man in the publication; and most of all did we anticipate benefit from the removal of the office of publication from the city of Buffalo to the great centre of our country. We looked upon medicine as national, and deemed that place the best for our journal where we would be able to command the most material for making it useful and practical. It was our hope and anticipation, at the commencement of the fifteenth volume, that after our journal had been firmly established, on its own basis, in New York, we would be able to form a union with one of the metropolitan journals, which would be for the good of both. Such a step would be so palpably for the benefit of those who honor us by their good-will, as to need little more than a simple announcement from us, if it had been taken when our engagements for the year had been fulfilled. Circumstances have rendered it necessary that this step should be taken at the present time, and an explanation is therefore demanded on our part.

Our readers will recollect an advertisement which appeared in our October number, for which we apologized, with the assurance that it should never again occur. We had been careful to retain the absolute power of excluding all improper advertisements, holding *ourselves* responsible personally for anything which appeared in any part of the Journal; and we had anticipated that the Buffalo publisher would see the policy—if he did not realize the degradation of another course—of keeping the Journal pure and unsullied. With that idea, we omitted to inspect all the advertisements before the work went to the bindery, and the objectionable one was thus suffered to appear. We were wrong in supposing that one who advertises quack remedies—female regulating pills, etc.—in the daily press, would be able to make a distinction, unless under compulsion, in favor even of the old Buffalo Medical Journal. This we became aware of when we forbade the insertion of any more such matter, and especially when our absence from the city was taken advantage of to smuggle in the same offensive circular in the January number. We were in Buffalo at the time of this last occurrence, engaged in the business which has resulted in the change now announced. Before this, we had supposed it possible to continue till the end of the volume under the then existing circumstances; though we saw the necessity of at that time purging the JOURNAL of an element so offensive to us and the profession. Seeing, however, the great injury daily suffered by the JOURNAL, we made up our mind that the publisher must be gotten rid of *at once;* and we therefore have done so. We are sorry to be thus forced to bring the private affairs of the JOURNAL into a place which should only be occupied by scientific matter; but we felt it our duty to let our subscribers know that we had never given up the right to sustain the character of the JOURNAL in all respects. That if our late publisher should in any way attempt to detract from the honor of the JOURNAL, to violate its chastity, we would *turn him out.* That *we* were not to be satisfied with disconnecting ourselves with such a publication as it would then be, but if necessary—and thank Heaven it is not necessary—we would play the part of the Roman father, and the publication should die.

As regards the present aspect of the JOURNAL, we desire to state that Mr. Mathews has no further connection with it whatever; nor is he connected with us in any way, as all business matters are now definitely closed. Mr. Mathews receives the subscriptions that may be paid on the *fourteenth* and *fifteenth* volumes; and our subscribers may look to us to supply them with the three numbers necessary to make the fifteenth volume complete. We do not receive anything from subscribers for the present volume, but at the *commencement of the next volume* subscriptions should be sent to Dr. J. H. DOUGLAS, Editor of the *American Medical Monthly and New York Review*, No. 12 Clinton Place, New York. The JOURNAL will contain eighty pages or more, instead of sixty-four; in consequence of which, no deduction will be made hereafter for advance payment, making the price $3 per year, to be paid in advance.

The combined Journal is now issued, edited by Dr. DOUGLAS and ourselves jointly; but, for the benefit of our subscribers, for the next three months, *three numbers* will be paged successively with the numbers of the NEW YORK REVIEW, which they have already received; the cover will remain the same, and they will have a separate index at the end of the volume, as usual. They will receive eighty pages a month, during this time, instead of sixty-four. Our arrangements being necessarily considerably modified, we are compelled to omit this month the lecture of Dr. DALTON. This, however, is only temporary, and their publication will be resumed.

We hope that the arrangements which have been made will be satisfactory to our own subscribers; and we know that they will be benefited by the change. Our list is large, and composed of those who, many of them, have received our monthly issue for fifteen years; we have obligations to such men which cannot be ignored, and we hope and trust that we may always be permitted to fulfill these obligations, and that any change which we make in the JOURNAL may be for the better. To the subscribers of the *American Medical Monthly* we present our humble duty, praying that they may be as well satisfied with our labors as our older friends. AUSTIN FLINT, JR.

— The editorial which DR. FLINT has addressed to the subscribers of the *Buffalo Medical Journal* will indicate to the readers of the MONTHLY the union of journals which has been accomplished since our last issue. In completing the consolidation therein referred to, we are materially enlarging the area of our own influence, and adding to the solid elements of our journal. In the accession of DR. FLINT and the collaborators of his journal to the host of good friends who have lent their valuable aid to the establishment of the MONTHLY, we see an abundant cause of congratulation to our readers, as well as to ourselves; and we hope hereafter, by our joint exertions in the field of medical journalism, to merit the continued support of our old friends, and to gain the esteem of our new ones.

